# Hydroxyethyl Cellulose as Water‐Soluble Co‐Binder for High Mass Loading LiNi_0.5_Mn_1.5_O_4_ Lithium‐Ion Battery Cathodes

**DOI:** 10.1002/cssc.202500079

**Published:** 2025-03-31

**Authors:** Qi Li, Matthias Kuenzel, Jian Wang, Thomas Diemant, Peter Axmann, Margret Wohlfahrt‐Mehrens, Stefano Passerini, Dominic Bresser

**Affiliations:** ^1^ Helmholtz Institute Ulm (HIU) 89081 Ulm Germany; ^2^ Karlsruhe Institute of Technology (KIT) 76021 Karlsruhe Germany; ^3^ Zentrum für Sonnenenergie-und Wasserstoff-Forschung 89081 Ulm Baden-Württemberg (ZSW Germany; ^4^ Transport Technologies Center Austrian Institute of Technology (AIT) Giefinggasse 2 1220 Vienna Austria; ^5^ Ulm University (UUlm) 89069 Ulm Germany

**Keywords:** aqueous processing, binder, LiNi_0.5_Mn_1.5_O_4_, cathode, lithium-ion battery

## Abstract

Combining high‐voltage cobalt‐free LiNi_0.5_Mn_1.5_O_4_ (LNMO) with fluorine‐free water‐soluble binders holds the promise of achieving more sustainable and environment‐friendly lithium‐ion batteries (LIBs). However, achieving high mass loading electrodes with lithium transition metal oxides as the active material remains a challenge. Herein, 2‐hydroxyethyl cellulose (HEC) is proposed as suitable binding agent, crosslinked *via* citric acid with guar gum (GG). The incorporation of HEC is pivotal for realizing a homogeneous dispersion of the electrode components, which is essential for the mechanical properties. Hence, the advantageous combination of co‐crosslinked HEC and GG allows for the simultaneous optimization of electrochemical and mechanical properties, enabling the preparation of well performing high mass loading LNMO electrodes with about 15 mg cm^−2^, providing a capacity retention as good as reference electrodes employing polyvinylidene difluoride as binder. Coupling these electrodes with graphite‐based negative electrodes enables lithium‐ion cells with an areal capacity of ~2.2 mAh cm^−2^ and a capacity retention of 82 % after 200 cycles, rendering this system promising for the realization of water‐processed, F‐free, high‐voltage cathodes.

## Graphical abstract

2‐Hydroxyethyl cellulose is introduced as water‐soluble co‐binder to achieve high electrode mass loadings for fluorine‐free LiNi_0.5_Mn_1.5_O_4_ cathodes. In combination with guar gum, the electrochemical and mechanical properties are simultaneously optimized, enabling graphite∥LiNi_0.5_Mn_1.5_O_4_ cells with an areal capacity of 2.2 mAh cm^‐2^ and a capacity retention of 82% after 200 cycles.

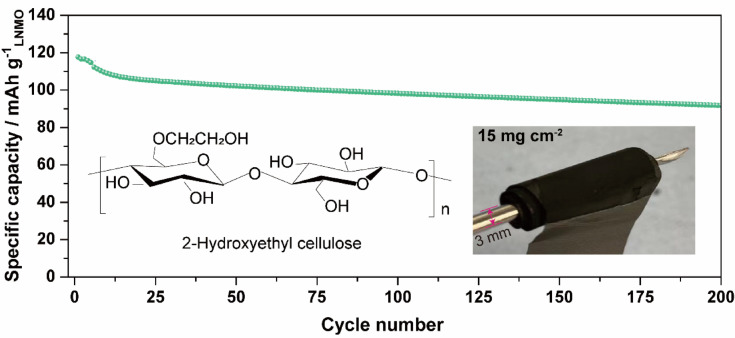

## Introduction

The demand for lithium‐ion batteries (LIBs) has been rapidly increasing over the past three decades due to their successful application in portable electronic devices, (hybrid) electric vehicles, and stationary energy storage systems.[^1^, ^2^, ^3^, ^4^] The current manufacturing of LIBs, specifically high‐energy LIBs, though, is commonly relying on the use of lithium transition metal oxides such as LiCoO_2_ or (Ni‐rich) LiNi_1‐x‐y_Co_x_Mn_y_O_2_ (NCM) as active materials for the positive electrode. Cobalt‐free and nickel‐poor LiNi_0.5_Mn_1.5_O_4_ (LNMO) is one of the most promising and more sustainable alternatives owing to its competitive energy density at the full‐cell level as a result of its high de‐/lithiation potential of ca. 4.7 V *vs*. Li^+^/Li and theoretical specific capacity of 147 mAh g^‐1^.[^5^, ^6^, ^7^, ^8^] While such replacement of the cathode active material will certainly have a great impact on the overall sustainability of LIBs, another very relevant factor is the choice of the binder (and the corresponding solvent) for the positive electrode. In fact, the current state of the art is still based on polyvinylidene difluoride (PVdF) as binder and *N*‐methyl‐2‐pyrrolidone (NMP) as solvent, both of them being harmful and toxic.[^9^, ^10^] The solvent can be avoided by transitioning to dry coating approaches, as recently reported Yao *et al*.,^[11]^ while the binder used therein, i. e., polytetrafluoroethylene binder (PTFE), still contained a high concentration of costly and environmentally harmful fluorine. The transition to water‐soluble, fluorine‐free bio‐derived binders such as sodium‐carboxymethyl cellulose (CMC), commonly used already for graphite‐based negative electrodes, promises not only a great step forward towards more sustainable LIBs, but may moreover enable lower cost and easier recycling.[^12^, ^13^, ^14^, ^15^, ^16^, ^17^] Following these considerations, many (bio‐derived), fluorine‐free and water‐soluble binding agents have been studied, including CMC, guar gum (GG), chitosan, alginate, and polyvinyl acetate, frequently providing results that are at least comparable to PVdF.[^18^, ^19^, ^20^, ^21^] Beside remaining challenges concerning a potential Li^+^/H^+^ exchange when lithium transition metal oxides get in contact with water, which is expected to be substantially lower for spinel‐structured LNMO, though, compared to layered oxides,^[22]^ the active material mass loading commonly remained (well) below 10 mg cm^−2^, while mass loadings twice to thrice higher are needed for commercial cells.[^23^, ^24^, ^25^] This is frequently very difficult for the reported rather simple binder compositions, though. For instance, using solely GG as binder makes it hard to further increase the areal loading of LNMO beyond 9 mg cm^−2^, owing to the high water content in the slurry that needs to be removed during the subsequent drying process, leading to insufficient mechanical properties of the resulting electrode tapes.^[26]^ Thus, novel binder compositions and alternative polymers are needed to push water‐soluble cathode compositions to the next level toward their potential commercialization. One possible alternative binder that has not attracted any attention in the field of battery research so far is 2‐hydroxyethyl cellulose (HEC). HEC has been reported to provide the capability of yielding highly homogeneous dispersions of carbon in cement.^[27]^ Such pronounced dispersion capability is considered essential for obtaining electrodes with suitable mechanical properties,^[28]^ which may eventually enable the achievement of high mass loading cathodes with suitable mechanical and electrochemical properties. Hence, we explored herein the combination of (citric acid crosslinked) GG and HEC as a novel binding agent for LNMO electrodes. The results show that the resulting high mass loading electrodes with ca. 15 mg cm^−2^ LNMO and an optimized GG:HEC ratio provide suitable mechanical and electrochemical properties comparable to reference electrodes containing PVdF.

## Results and Discussion

**Figure 1:**
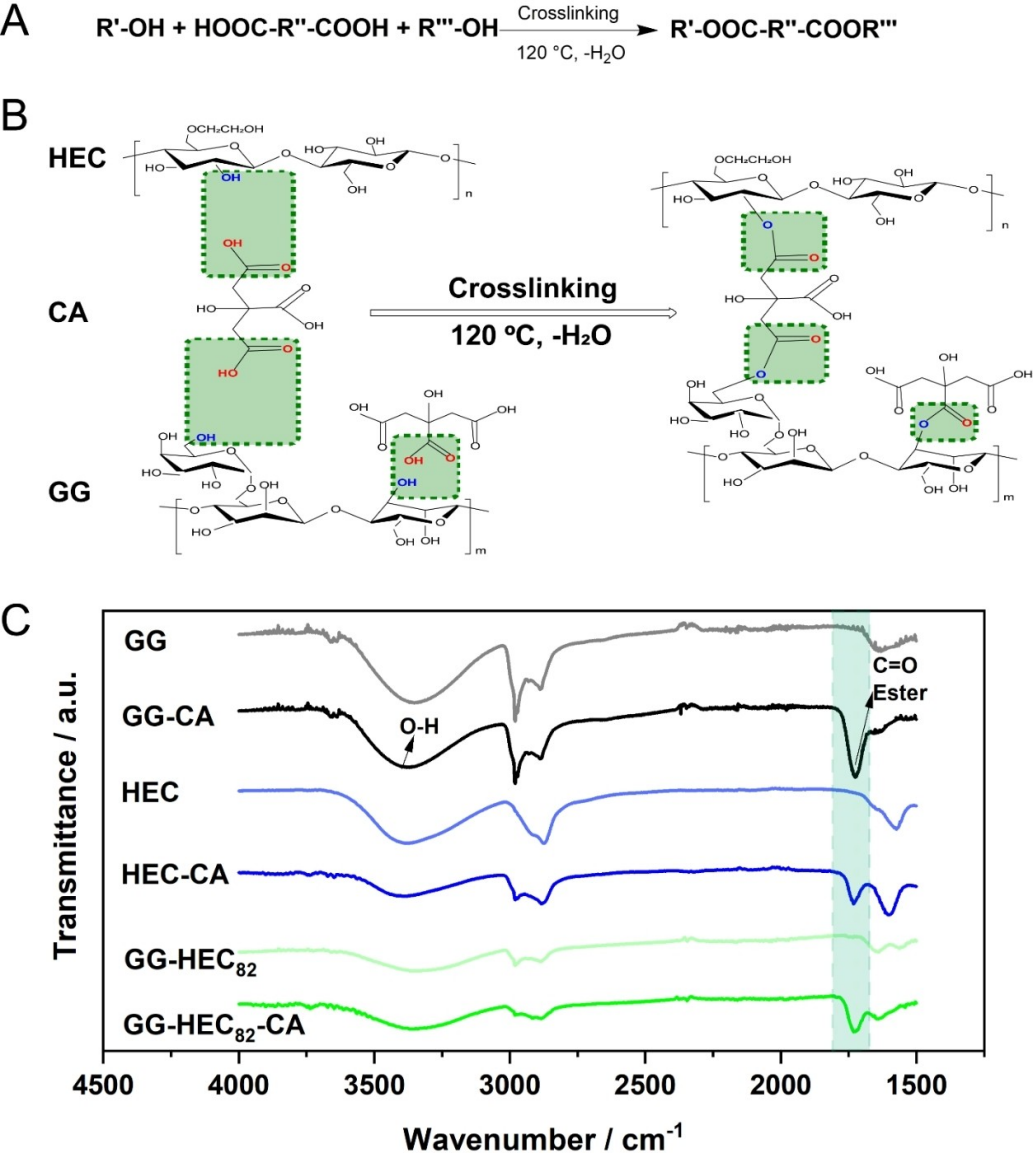
(A) Chemical equation of the esterification reaction. (B) Schematic illustration of the CA‐induced crosslinking reaction involving GG and HEC. (C) FT‐IR spectra of GG, GG‐CA, HEC, HEC‐CA, GG‐HEC_82_, and GG‐HEC_82_‐CA (from top to bottom). The wavenumber region of the C=O stretching vibration of the ester group (ṽ=1726 cm^−1^) is marked with a green background.

The CA‐induced crosslinking reaction between GG and HEC is depicted in Figure 1A,B according to previous studies on, amongst others, CMC and GG.^[29]^ A robust binder network with high mechanical strength is key to resist any electrode delamination during the process of cell fabrication or operation. In detail, the hydroxyl groups (−OH) of GG and HEC are expected to react with the carboxyl groups (−COOH) of CA to form ester bonds when the temperature reaches ca. 120 °C. As a result, GG and HEC are expected to be interconnected by the bridged CA.[^30^, ^31^, ^32^, ^33^] In fact, there are abundant –OH groups present in both GG and HEC, though presumably more –OH groups in GG will react with –COOH in CA compared to HEC (marked in light green) owing to the lower reactivity of the –OH present in HEC.^[34]^ The detailed esterification reaction scheme is presented in Figure 1B, and the successful reaction via esterification was confirmed by Fourier‐transform infrared (FT‐IR) spectroscopy (Figure 1C). The given spectra of neat GG, HEC and GG‐HEC_82_ (GG:HEC=8 : 2 (*w*/*w*)) samples were used as references. Generally, the broad band at a wavenumber (ṽ) of 3398 cm^−1^ can be assigned to the hydroxyl (−OH) stretching vibration, which exists in all these samples. However, a new band occurs for the spectra of GG‐CA, HEC‐CA, and GG‐HEC_82_‐CA at approximately ṽ=1726 cm^−1^, which is the characteristic C=O stretching vibration of ester groups (marked in green), corroborating the proposed crosslinking reaction for HEC and GG.

As mentioned in the introduction, HEC was introduced to enhance the dispersion of the electrode components, especially the nanoparticulate conductive carbon, which is essential for achieving suitable mechanical properties.[^27^, ^28^, ^35^] This was investigated by scanning electron microscopy (SEM) and energy‐dispersive X‐ray spectroscopy (EDX). In a first step, we prepared electrodes comprising the binder and conductive carbon only to get a first idea of the carbon distribution. The comparison of the three different combinations, i. e., GG‐CA+C, HEC‐CA+C, and GG‐HEC_82_‐CA+C, presented in Figure S1, reveals that the conductive carbon forms some rather large agglomerates in the case of GG‐CA+C (Figure S1A), while it is homogeneously distributed in HEC‐CA+C (Figure S1B) and GG‐HEC_82_‐CA+C (Figure S1C). These findings highlight the beneficial impact of HEC on the carbon distribution as earlier reported for cement.^[27]^

**Figure 2:**
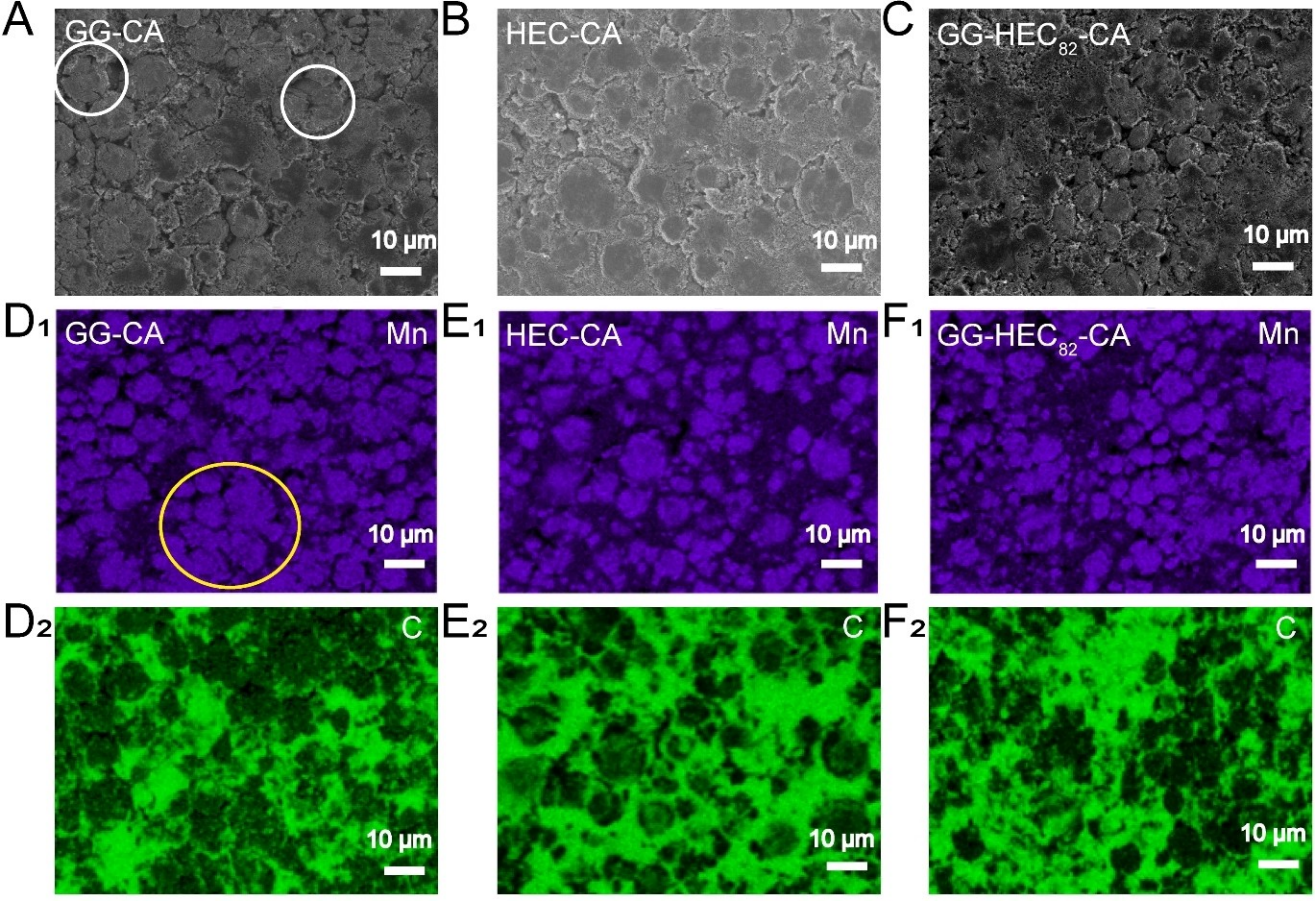
Comparison of the SEM micrographs and the corresponding EDX mappings of manganese and carbon for (A, D_1_, D_2_) GG‐CA, (B, E_1_, E_2_) HEC‐CA, and (C, F_1_, F_2_) GG‐HEC_82_‐CA‐based electrodes before cycling. Some minor cracks and agglomerates are highlighted by colored circles.

In the next step, we extended this investigation to LNMO‐based electrodes. The results of the SEM analysis are presented in Figure 2A‐2 C. Figure 2A reveals that there are some minor cracks in the electrode coating layer of the GG‐CA‐based electrode (marked by white circles), while there are no cracks observed for the HEC‐CA (Figure 2B) and GG‐HEC_82_‐CA‐based electrodes (Figure 2C). We may assume that the crack formation is the result of a somewhat more inhomogeneous LNMO and carbon distribution, as observed *via* EDX mapping of manganese (Figure 2D_**1**_) and carbon (Figure 2D_**2**_). In fact, the dispersion of the LNMO and carbon particles in the HEC‐CA (Figure 2E_**1**_,E_2_) and GG‐HEC_82_‐CA‐based electrodes (Figure 2F_**1**_,F_2_) appears more homogeneous, i. e., without any larger LNMO clusters. Once again, the incorporation of HEC, even with a relatively low amount, leads to a better electrode material dispersion, just as observed before.

**Figure 3:**
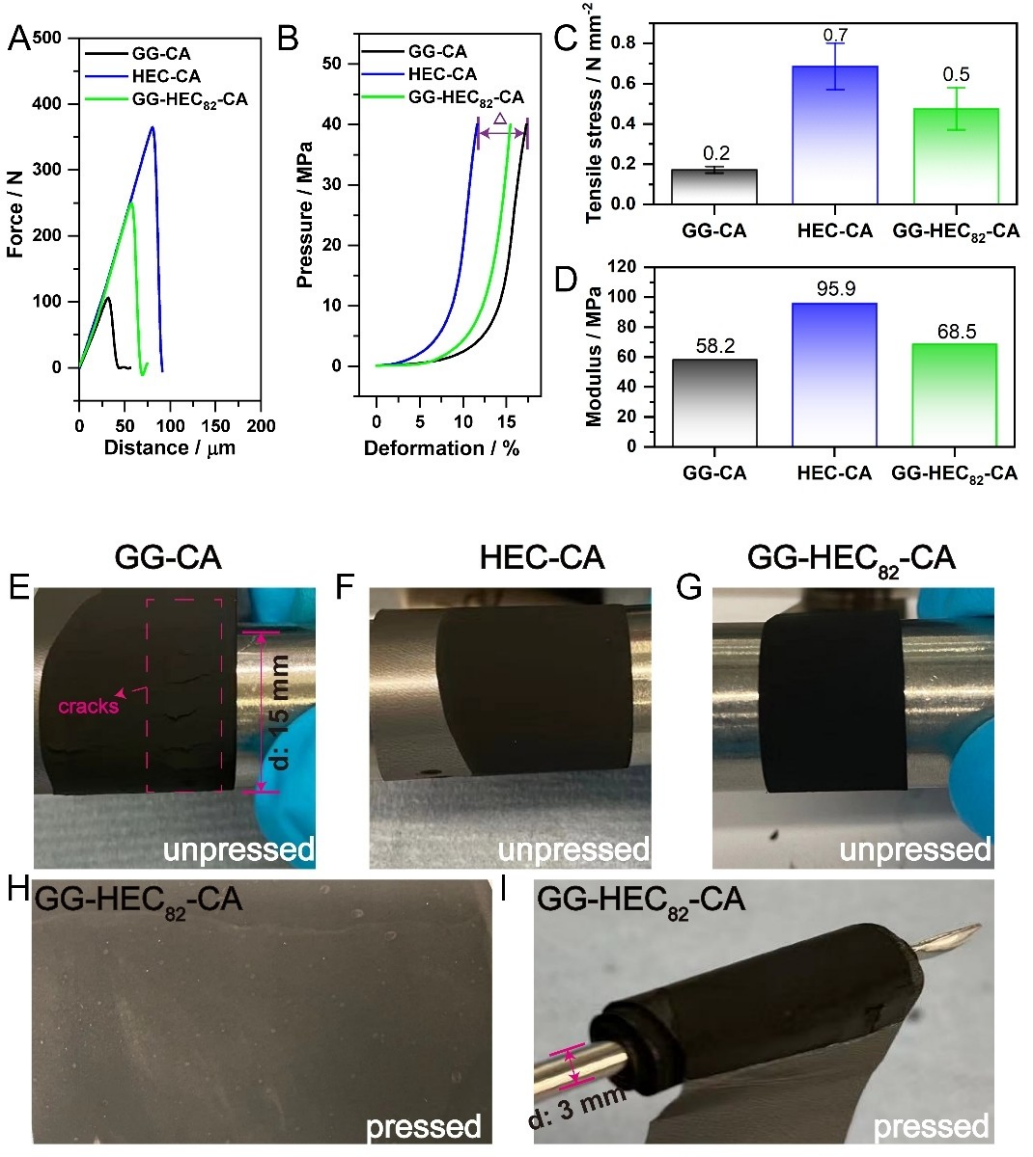
Data obtained from (A) the peel test and (B) the compression test for GG‐CA (black), HEC‐CA (blue), and GG‐HEC_82_‐CA‐based electrodes (green) after calendaring. (C) Tensile stress calculated from the data presented in (A). (D) Modulus obtained from the data presented in (B). Photographs of the different non‐pressed electrode tapes during the bending test: (E) GG‐CA, (F) HEC‐CA, and (G) GG‐HEC_82_‐CA‐based electrodes. (H) Photograph of the GG‐HEC_82_‐CA‐based electrode after calendaring. (I) Photograph of the calendared GG‐HEC_82_‐CA‐based electrode wound around a thin spatula.

To further investigate the impact of using HEC, we evaluated the mechanical properties of these three different electrodes, which is an important parameter when studying new binder systems, as these are essential for maintaining electrode integrity during the electrode preparation and processing.[^36^, ^37^, ^38^] We carried out peel tests to check the adhesion and cohesion strength of the as‐prepared electrode tapes.^[39]^ The experimental setup is schematically depicted in Figure S2A,B and Figure S2C shows a photo of the tested electrodes, demonstrating that the detachment occurred mainly within the coating layer, indicating that the cohesion within the coating layer is weaker than the adhesion of the coating layer to the aluminum current collector. The data obtained are displayed in Figure 3A. It is easy to note that the force to peel off the HEC‐CA‐based electrode is stronger compared to the GG‐HEC_82_‐CA and GG‐CA‐based electrodes. Figure 3C further compares the tensile stress (maximum force per unit area) upon peeling, which shows quantitatively that 0.2 N mm^−2^ is sufficient to detach the GG‐CA electrode coating layer. This is less than one third of the HEC‐CA‐based electrodes with 0.7 N mm^−2^, and less than half compared to the GG‐HEC_82_‐CA‐based electrodes with 0.5 N mm^−2^. Once again, even adding only a minor amount of HEC has a relatively large impact on the mechanical properties, substantially increasing the adhesion of the coating layer to the current collector.

In addition, compression tests were carried out to investigate the elasticity of the different electrode types to get an idea of the capability of these electrodes to withstand mechanical stress. Figure 3B shows the data obtained *via* the compression tests, where the deformation, i. e., the ratio of the change in thickness compared to the original thickness, as a result of the pressure applied is displayed. For GG‐CA, the deformation is much larger than in the case of HEC‐CA and GG‐HEC_82_‐CA at the same force. This is also reflected by the corresponding moduli, being the lowest for GG‐CA with about 58.2 MPa compared to the other two kinds of electrodes with about 68.5 MPa for GG‐HEC_82_‐CA and 95.9 MPa for HEC‐CA (Figure S3 and Figure 3D). Hence, the ability to tolerate volume fluctuations is greater when containing HEC, meaning that the cohesion within the electrode coating layer is superior.

These findings concerning the mechanical properties of the electrode layers are also apparent from a rather qualitative experiment, illustrating the impact upon electrode processing, in which the (non‐calendared nor pressed) electrodes were wound around a metallic cylinder (Figure 3E‐3G). In the case of GG‐CA, some cracks are observed even by eye (Figure 3E), while there are no apparent cracks for HEC‐CA (Figure 3F) and GG‐HEC_82_‐CA (Figure 3G). Also for calendared electrodes, the GG‐HEC_82_‐CA‐based electrodes still show a smooth and defect‐free surface morphology (Figure 3H), which is even maintained when winding the electrode around a thin spatula rather than a thicker metallic cylinder (Figure 3I).

**Figure 4:**
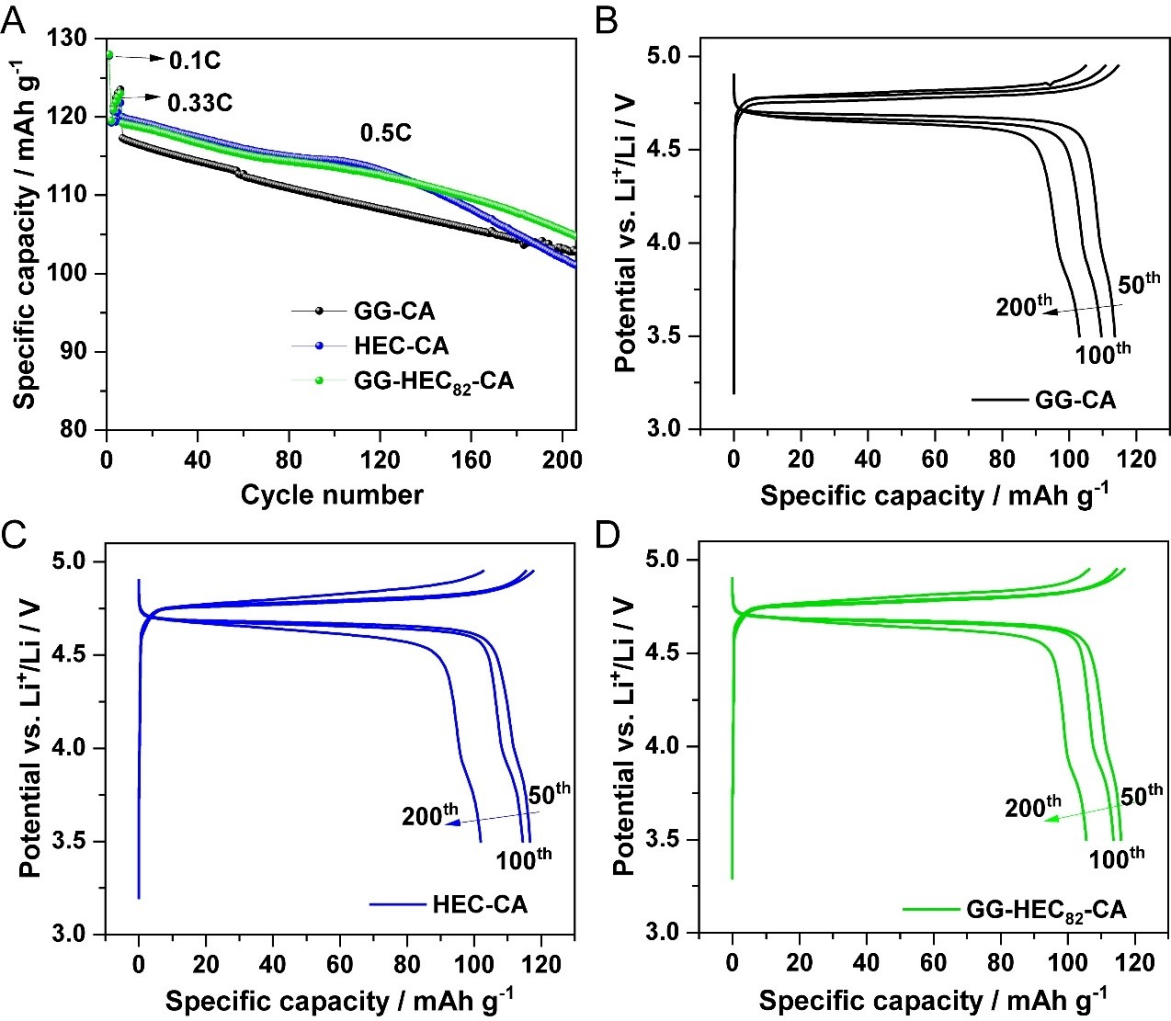
Galvanostatic cycling of Li∥LNMO cells containing GG‐CA (black), HEC‐CA (blue) and GG‐HEC_82_‐CA (green) as binder at 0.5 C after one formation cycle at 0.1C and five cycles at 0.33 C: (A) Specific discharge capacity as a function of cycle number. (B−D) Selected dis‐/charge profiles (50^th^, 100^th^, 200^th^) for (B) GG‐CA, (C) HEC‐CA, and (D) GG‐HEC_82_‐CA. The cut‐off potentials were set to 3.5 and 4.95 V *vs*. Li^+^/Li (T=20 °C).

Following this comprehensive investigation of the electrode composition and their mechanical properties, we studied their electrochemical properties by subjecting them to galvanostatic cycling in half‐cell configuration, i. e., with a lithium‐metal counter electrode. A comparison of the three different electrodes is presented in Figure 4A. In all three cases, there is a certain decrease in capacity upon cycling for more than 200 cycles. Please note the given scale of the y axis, though, which we chose to highlight the differences for the three binding systems. Interestingly, HEC‐CA and GG‐HEC_82_‐CA show higher specific discharge capacities than GG‐CA, which might be attributed to the inferior carbon distribution in the case of GG‐CA, i. e., a less effective electronic wiring in the electrode coating layer, and/or an incomplete accessibility of the LNMO active material in the latter case owing to the less homogeneous electrode composition and crack formation observed earlier. Nonetheless, after about 110 cycles the HEC‐CA‐based electrodes start fading a little faster than cells with the other two electrode types, though still providing a specific capacity of 101 mAh g^−1^ after 200 cycles, which is only slightly lower than the capacity recorded for GG‐CA with about 103 mAh g^−1^. The latter and GG‐HEC_82_‐CA remain running in parallel essentially, with GG‐HEC_82_‐CA maintaining a higher capacity by about 2–3 mAh g^−1^ throughout the experiment; in fact, starting at 120 mAh g^−1^ (GG‐HEC_82_‐CA) and 117 mAh g^−1^ (GG‐CA). As a result, both kinds of cells show a capacity retention of ca. 88 % after 200 cycles at 0.5C. This finding confirms that the benefits of both polymers can be combined when simply blending them in an appropriate ratio. The very similar behavior of GG‐CA and GG‐HEC_82_‐CA is further reflected in a very comparable evolution of the corresponding dis‐/charge profiles with regard to capacity fading and increase in overpotential (Figure 4B‐4D). In the case of HEC‐CA, there is a slightly larger increase in overpotential observed for the 200^th^ cycle, which is in line with the more rapid fading after about 110 cycles, indicating that there are some side reactions occurring upon long‐term cycling, which leads to an increase in interfacial resistance.[^40^, ^41^]

**Figure 5:**
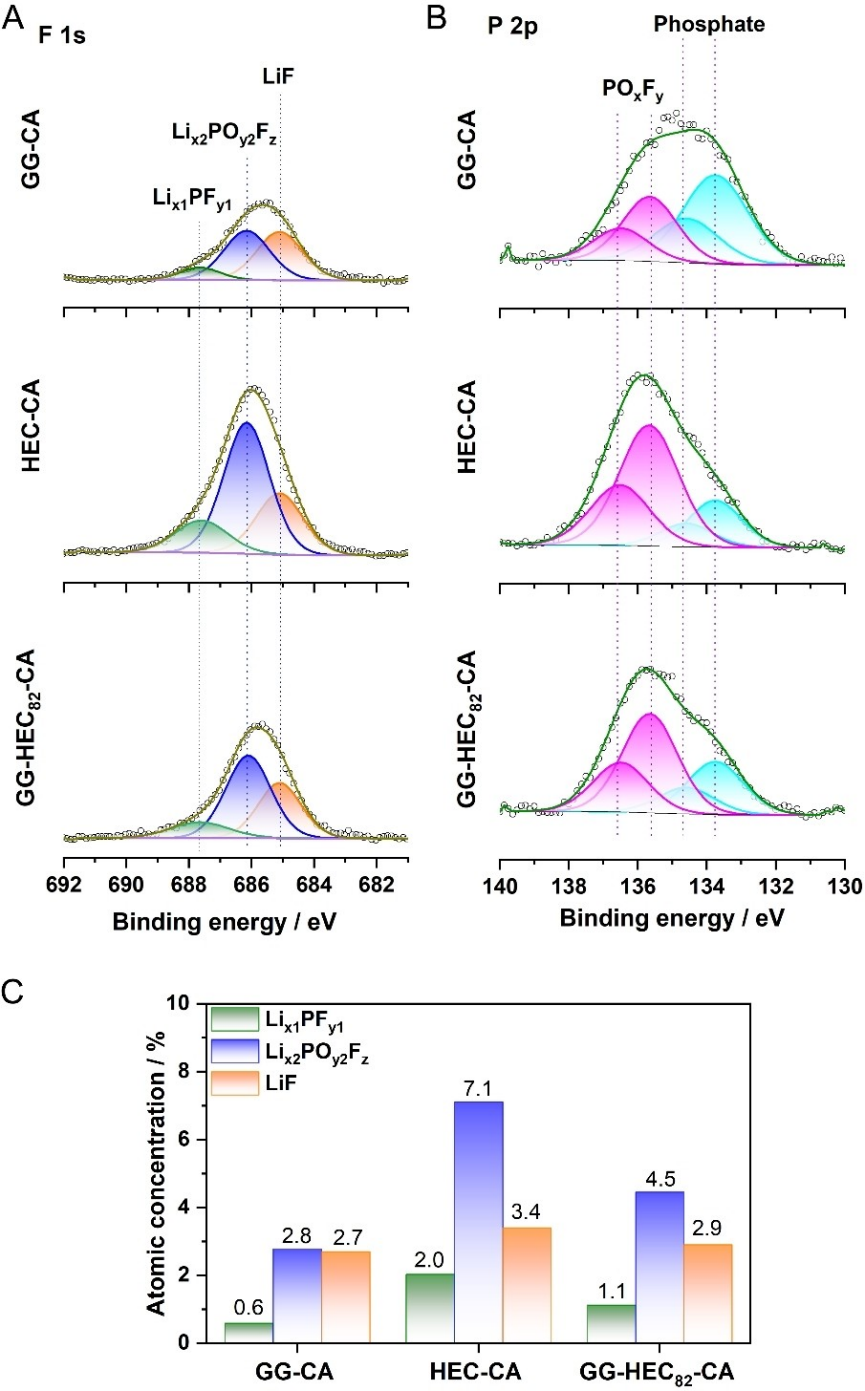
Comparison of the *ex situ* XPS data recorded for GG‐CA, HEC‐CA, and GG‐HEC_82_‐CA‐based electrodes cycled in Li∥LNMO cells for 300 cycles at 0.5C: Detail spectra in the (A) F 1s and (B) P 2p region. (C) F atomic concentrations in the corresponding F‐containing compounds as determined by quantitative analysis.

To gain a better understanding of these side reactions, especially concerning the decomposition of the lithium salt LiPF_6_ in the electrolyte, we conducted *ex situ* X‐ray photoelectron spectroscopy (XPS) measurements. The results of the detailed measurements in the F 1s and P 2p regions are displayed in Figure 5. Focusing first on the F 1s spectra (Figure 5A), the three peaks at 685.1, 686.2, and 687.6 eV were detected for all three electrode formulations, which, can be assigned to LiF, Li_x2_PO_y2_F_z_, and Li_x1_PF_y1_, respectively.[^42^, ^43^] Figure 5C shows the atomic concentrations of these F species, as determined by quantitative analysis of the detail spectra. Comparison of the results shows a significantly larger concentration of all LiPF_6_ decomposition products in the surface layer of the HEC‐CA than in the GG‐CA electrode, while the GG‐HEC_82_‐CA electrode is in‐between. Next, the P 2p spectra were analyzed (Figure 5B). Two sets of P 2p peak doublets were employed, which can be attributed to (1) Li_x2_PO_y2_F_z_ and Li_x1_PF_y1_ species (P 2p_3/2_ peak at 135.7 eV) and (2) phosphates (P 2p_3/2_ peak at 133.5 eV), either as a remnant of phosphoric acid addition during electrode preparation or coming from complete decomposition of LiPF_6_.^[44]^ It should be mentioned that LiPF_6_ itself is not present in the surface layer of the electrodes, since its peak doublet was not detected (P 2p_3/2_ expected above 137 eV). By comparing the intensity of the Li_x2_PO_y2_F_z_/ Li_x1_PF_y1_ feature for GG‐CA, HEC‐CA, and GG‐HEC_82_‐CA, it is apparent that it decreases in the order HEC‐CA > GG‐HEC_82_‐CA > GG‐CA, which is consistent with the results observed in the F 1s spectra. Taken together, these findings corroborate an increased LiPF_6_ decomposition in the case of HEC, which provides an explanation for the pronounced fading compared to GG‐CA and GG‐HEC_82_‐CA, while this can be effectively suppressed in the latter case by blending it with GG.

To further evaluate the potential of GG‐HEC_82_‐CA as binder system for high mass loading cathodes, we conducted a direct comparison with PVdF as the current state‐of‐the‐art binder (Figure S4). The cells containing GG‐HEC_82_‐CA as binder generally showed a slightly lower capacity, which might result from the initial reaction with water and the two acids added to the slurry,[^46^, ^47^] but the overall cycling stability is essentially the same with a capacity retention of about 90 % in both cases after 200 cycles at 0.5C (Figure S4A), which is also reflected in a very similar evolution of the dis‐/charge profiles upon cycling (Figure S4B).

**Figure 6:**
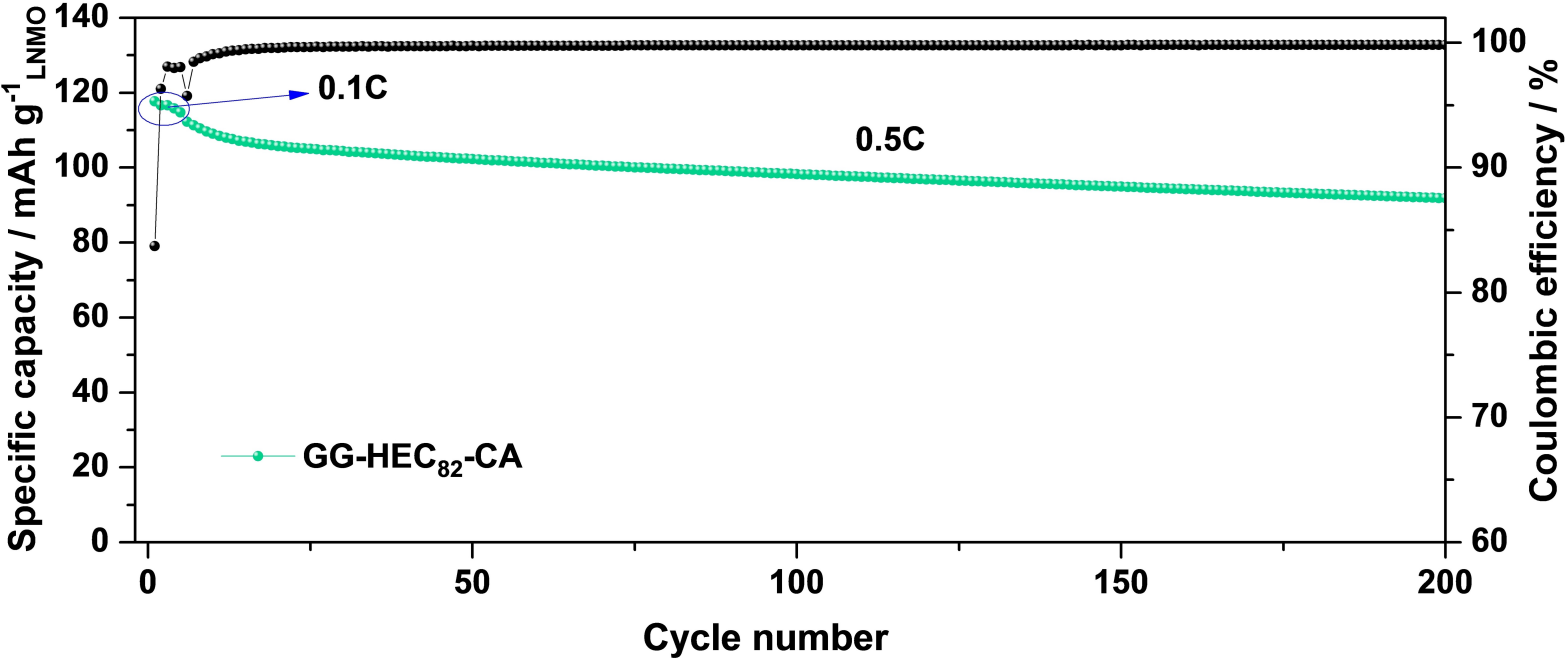
Galvanostatic cycling of graphite∥LNMO lithium‐ion cells containing GG‐HEC_82_‐CA as binder for the LNMO electrodes and CMC as binder for the graphite electrodes at 0.5C after five formation cycles at 0.1C. The N/P ratio was about 1.15 and the cut‐off voltages were set to 2.6 and 4.92 V (T=20 °C; 1C=147 mA g^−1^).

Finally, we assembled graphite∥LNMO full‐cells with an areal capacity of ~2.2 mAh cm^−2^ to further assess the feasibility of employing GG‐HEC_82_‐CA (and, especially HEC) as binder for commercial lithium‐ion cells (Figure 6). Following the state of the art for graphite‐based electrodes and to yield a lithium‐ion cell that is as sustainable as possible with the given cell chemistry, we used CMC and styrene‐butadiene rubber (SBR) as binder for the negative electrodes. The initial reversible capacity of these full‐cells was 117 mAh g^−1^ at 0.1C and the first‐cycle Coulombic efficiency was 83.7 %. This value is in relatively good agreement with previous studies on this electrode combination, though there is certainly still room for improvement, e. g., by optimizing the electrolyte composition.[^48^, ^49^, ^50^] After 200 cycles at 0.5C, the capacity retention of this lab‐scale lithium‐ion full‐cell was 82 %, i. e., above the common threshold of 80 % for the end of life.

## Conclusions

2‐Hydroxyethyl cellulose (HEC) is proposed as co‐binder to enhance the mechanical properties of high mass loading LiNi_0.5_Mn_1.5_O_4_ (LNMO) electrodes, which leads to an improved cohesion within the coating layer and its adhesion to the current collector, as revealed *via* peel and compression tests, by enabling a more homogeneous dispersion of the conductive carbon. These enhanced mechanical properties, however, come at the expense of a more pronounced LiPF_6_ decomposition, resulting in a faster capacity fading upon long‐term cycling. Blending HEC with guar gum (GG) in an optimized ratio of 2 : 8 allows for overcoming this challenge while maintaining the benefits. The resulting electrodes offer a capacity retention that is essentially the same as for LNMO electrodes containing polyvinylidene difluoride as the current state of the art binder. Furthermore, they provide stable cycling also in fully aqueous processed graphite∥LNMO lithium‐ion cells, thus, highlighting the potential use in commercial cells.

## Experimental Section

### Electrode Preparation

The positive electrodes had a composition of 92 wt.% LiNi_0.5_Mn_1.5_O_4_ (LNMO; ZSW), 5 wt.% carbon black (C‐Energy Super C65, IMERYS), and 3 wt.% of the binder. A detailed description of the synthesis of the LNMO active material has been reported by Axmann *et al*.^[51]^ The binding agents and other electrode components used herein were guar gum (GG; Lamberti SpA), 2‐hydroxyethyl cellulose (HEC; M_w_=380,000 g mol^−1^, d: 0.6 g ml^‐1^, Sigma‐Aldrich), citric acid (CA; 99 %, Sigma‐Aldrich), and orthophosphoric acid (PA; 85 wt.%, Bernd Kraft) diluted to 20 wt.%. If not indicated differently, CA and PA were always added to the electrode mixture in order to crosslink the cellulose‐type polymers and adjust the pH value. In detail, the weight ratio between the polymer binders and CA was 9 : 1, and the PA content was 0.3 wt.% with regard to the mass of LNMO. The recipes of the aqueous binder solutions are listed as follows:

1. GG‐HEC_91_, GG‐HEC_82_, or GG‐HEC_73_ binder solutions: mixtures of guar gum (GG) and 2‐hydroxyethyl cellulose (HEC) with different weight ratios of 9 : 1, 8 : 2, and 7 : 3 were dissolved in deionized water to obtain 1.5 wt.% solutions.
2. GG‐CA binder solution: the mixture of GG and CA was dissolved in deionized water to obtain a 1.5 wt.% solution.
3. HEC‐CA binder solution: the mixture of HEC and CA was dissolved in deionized water to obtain a 3.2 wt.% solution.
4. GG‐HEC_82_‐CA binder solution: the weight ratio of GG and HEC was 8 : 2, and the mixture of GG, HEC and CA was dissolved in deionized water to obtain a 1.8 wt.% solution.
5. In addition, PVdF‐based electrodes were prepared as reference. The electrodes had a composition of 92 wt.% LNMO, 5 wt.% conductive carbon (C‐Energy Super C65, IMERYS), and 3 wt.% polyvinylidene fluoride (PVdF, Solef6020, Solvay). The binder was dissolved in *N*‐methyl‐2‐pyrrolidone (NMP, Sigma‐Aldrich) to obtain a 5 wt.% solution.

For the Fourier‐transform infrared spectroscopy (FT‐IR) analysis, the binder solutions of GG, GG‐CA, HEC, HEC‐CA, GG‐HEC_82_, and GG‐HEC_82_‐CA were cast on Mylar foil. The wet films were then pre‐dried at 80 °C under ambient atmosphere and further dried at 120 °C under vacuum. The FT‐IR analysis itself was performed using a PerkinElmer UATR Two instrument within the range of 4000 to 400 cm^−1^.

For the preparation of the LNMO electrodes, the slurries containing all components were mixed using a Thinky mixer (ARE‐250) for 1 h at 2,000 rpm. The homogenized slurries were cast on carbon‐coated aluminum foil (thickness: 20 μm; battery grade) utilizing a laboratory‐scale doctor blade. The mass loading of the LNMO electrodes was in the range from 14 to 17 mg cm^‐2^. The initially obtained wet films were pre‐dried in an atmospheric oven (ED‐115, Binder) at 80 °C for 30 min and then transferred into a dry room (dew point of less than −70 °C) and stored there overnight. Disk‐shaped electrodes with a diameter of 12 mm were punched and pressed at 8 t for 10 s (Atlas manual hydraulic press, Specac) in the dry room. After that, the electrodes were further dried under vacuum at 120 °C for 14 h.

The mixtures with the three different mass ratios of GG and HEC, i. e., 9 : 1, 8 : 2, and 7 : 3, hereinafter referred to as GG‐HEC_91_, GG‐HEC_82_, and GG‐HEC_73_, respectively, were investigated to get a first idea for an optimum composition. The results are presented in Figure S5. Figure S5A shows that the GG‐HEC_82_‐based electrodes exhibit the best cycling stability and capacity retention (85 %) at 1C after 150 cycles compared to GG‐HEC_91_ with 82 % and GG‐HEC_73_ with 78 %, while all cells start the 1C cycling with essentially the same specific capacity. Interestingly, a higher overpotential evolves upon cycling in the case of the highest HEC content (GG‐HEC_73_, Figure S5B‐5D), while the capacity evolution follows a largely parallel trend for GG‐HEC_91_ and GG‐HEC_82_. Since GG‐HEC_82_ shows overall the best cycling stability and capacity retention, this composition (plus CA‐induced crosslinking) was chosen for all following tests and studies. The wet film thickness was set to 450 μm, while it has been 450 μm for GG‐CA and 300 μm for HEC‐CA to yield comparable active material mass loadings.

The morphology and composition distribution of the electrodes were studied *via* scanning electron microscopy (SEM; Zeiss crossbeam 340) equipped with an energy‐dispersive X‐ray spectrometer (EDX; Oxford instruments X–Max50, 50 mm^2^, 15 kV). For this purpose, also electrodes containing conductive carbon and the different binders only were prepared, adapting the same processing as for the LNMO‐containing electrodes.

For the full‐cell tests, the graphite‐based anodes had a composition of 94 wt.% graphite (SLP30, TIMCAL), 4 wt.% binder, and 2 wt.% conductive carbon (C‐Energy Super C45, IMERYS). The binder used for the anodes was a mixture of sodium carboxymethyl cellulose (CMC; Walocel CRT2000 GA 07, Dow Wolff Cellulosics) and styrene‐butadiene rubber (SBR; Wellcos BM‐451B) in a 1 : 1 weight ratio. Firstly, we prepared 3 wt.% CMC and 40 wt.% SBR water solutions that were combined to yield the aforementioned weight ratio. Subsequently, the graphite and the conductive carbon were added and the resulting slurry was homogenized using a Thinky mixer. The wet film thickness of the electrodes was set to 140 μm. The drying procedure was the same as for the cathodes. Eventually, the electrodes were pressed at 1 t for 10 s. The mass loading of graphite was balanced according to an N : P ratio of 1.15.

### Mechanical Characterization

Peel and compression tests were carried out using a ZwickRoell Allroundline testing machine following a procedure that has been reported by Haselrieder et al.^[39]^ Prior to the tests, electrode tapes with the same mass loading were pressed to a thickness of 115–125 μm *via* calendaring (MX 1051768, Saueressig). For the peel tests, the calendared electrode tapes were cut into several squared samples with an area of 6.45 cm^2^ and then fixed to the sample holder with adhesive tapes (Tesafix 5696). The movable planar plate, along with the adhesive tapes, was pressed against the fixed electrode tapes until the compressive loading reached 2500 N. After 30 s, the movable plate began to peel off the electrode tape at a velocity of 100 mm min^−1^. The maximum tensile force during peeling was recorded as the peeling force. The tensile stress ($\sigma_{n}$ ) is calculated by:

(1) $$\sigma_{n}=\frac{F_{max}}{A}$$

with $F_{max}$ being the maximum force and A being the area of the electrode tested.

For the compression tests, a 5 mm^2^ flat tip was pressed against the electrode until the pre‐load reached 0.5 N, which was the force required to make the electrode flat without inducing any deformation. The thickness of the electrode at this point was noted as the initial thickness ′*h_0_* ′. Then the flat tip continued to apply pressure to the electrode at a rate of 0.5 mm min^−1^ until the pressure reached 200 N. After releasing the loading completely, the machine recorded the recovered thickness of the electrode as ′*h_1_* ′. The elasticity of the electrode was expressed in terms of the deformation ratio, denoted as a percentage (%), calculated using the following equation:

(2) $$x=\frac{h_{0}-\;h_{1}}{h_{0}}*\;100$$

Multiple measurements were conducted by selecting different regions on each electrode, as represented by the error bars.

### Cell Assembly and Electrochemical Characterization

The electrochemical characterization was conducted using three‐electrode Swagelok‐type cells, which were assembled in an argon‐filled glovebox (MB200B ECO, MBraun) with a H_2_O and O_2_ content of less than 0.1 ppm. Lithium metal foil with a thickness of 500 μm (battery grade, Honjo) or graphite electrodes were chosen as counter electrodes, while lithium metal served as reference electrode. Glass fiber sheets (Whatman GF/D) were used as separator and soaked with 130 μL of the electrolyte solution (1 M LiPF_6_ in ethylene carbonate (EC): dimethyl carbonate (DMC), 1 : 1 (*wt*/*wt*), LP30, UBE). Galvanostatic cycling was conducted at 20 °C using a Maccor Battery Tester 4300. The cut‐off potentials were set to 3.5 V and 4.95 V (*vs*. Li^+^/Li) for the Li∥LNMO cells, and a C rate of 1C corresponds to a specific current of 147 mA g^‐1^. For the graphite∥LNMO cells, the cut‐off voltages were set to 2.6 and 4.92 V.

### Ex situ X‐Ray Photoelectron Spectroscopy

The cycled cells were disassembled in an argon‐filled glove box, and the electrodes were rinsed with DMC before being transferred to the load lock of the XPS system in an argon‐filled vessel. The measurements were performed using a Specs XPS system with monochromatized Al Kα radiation (1486.6 eV) and pass energies of 90 and 30 eV at the analyzer for the survey and detail measurements, respectively. Before peak analysis, the main C 1s peak of C−C/C=C species was set to 284.8 eV for energy calibration. The CasaXPS software was used for the fitting, entering Shirley‐type backgrounds and peaks with Gaussian‐Lorentzian shape.

## Conflict of Interests

The authors declare that there is no conflict of interest.

1

## Supporting information

As a service to our authors and readers, this journal provides supporting information supplied by the authors. Such materials are peer reviewed and may be re‐organized for online delivery, but are not copy‐edited or typeset. Technical support issues arising from supporting information (other than missing files) should be addressed to the authors.

Supporting Information

## Data Availability

The data that support the findings of this study are available from the corresponding author upon reasonable request.
