# Supplementary material for: Hydroxyethyl Cellulose as Water‐Soluble Co‐Binder for High Mass Loading LiNi0.5Mn1.5O4 Lithium‐Ion Battery Cathodes
Source: ChemSusChem. 2025 Mar 31;18(11):e202500079. doi: 10.1002/cssc.202500079 (PMC12131669; doi:10.1002/cssc.202500079)
Supplement: Supplementary file 1 — Supporting Information [file CSSC-18-e202500079-s001.pdf]

ChemSusChem

Supporting Information

Hydroxyethyl Cellulose as Water-Soluble Co-Binder for High Mass Loading $LiNi_{0.5}Mn_{1.5}O_4$ Lithium-Ion Battery Cathodes

Qi Li, Matthias Kuenzel, Jian Wang, Thomas Diemant, Peter Axmann, Margret Wohlfahrt-Mehrens, Stefano Passerini,* and Dominic Bresser*

Supporting Information

Hydroxyethyl Cellulose as Water-Soluble Co-Binder for High Mass Loading $LiNi_{0.5}Mn_{1.5}O_4$ Lithium-Ion Battery Cathodes

Qi Li,[1,2] Matthias Kuenzel,[1,2] Jian Wang,[1,2] Thomas Diemant,[1,2] Peter Axmann,[3] Margret Wohlfahrt-Mehrens,[3] Stefano Passerini[1,2,4,*] and Dominic Bresser[1,2,5,*]

[1]*Helmholtz Institute Ulm (HIU) 89081 Ulm, Germany*

[2]*Karlsruhe Institute of Technology (KIT), 76021 Karlsruhe, Germany*

[3]*Zentrum für Sonnenenergie- und Wasserstoff-Forschung Baden-Württemberg (ZSW), 89081 Ulm, Germany*

[4]*Austrian Institute of Technology (AIT), Transport Technologies Center, 1220 Vienna, Austria*

[5]*Ulm University (UUlm), 89069 Ulm, Germany*

Keywords: aqueous processing; binder; $LiNi_{0.5}Mn_{1.5}O_4$; cathode; lithium-ion battery

***Corresponding authors**: dominic.bresser@kit.edu; stefano.passerini@kit.edu

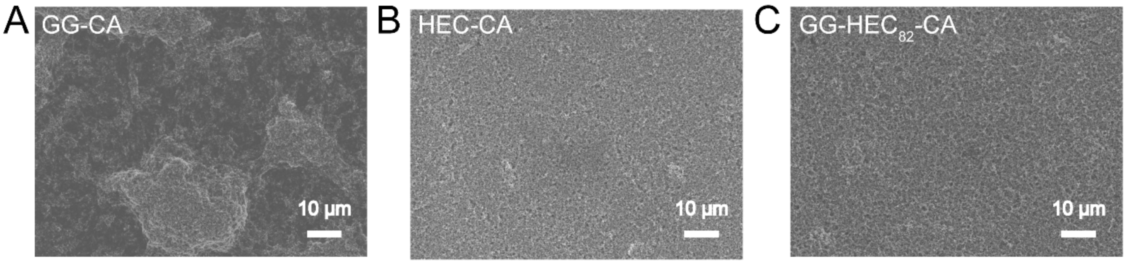

Figure S1. SEM micrographs of unpressed electrodes consisting of conductive carbon and the three different binders: (A) GG-CA, (B) HEC-CA, and (C) GG-HEC_{82}-CA.

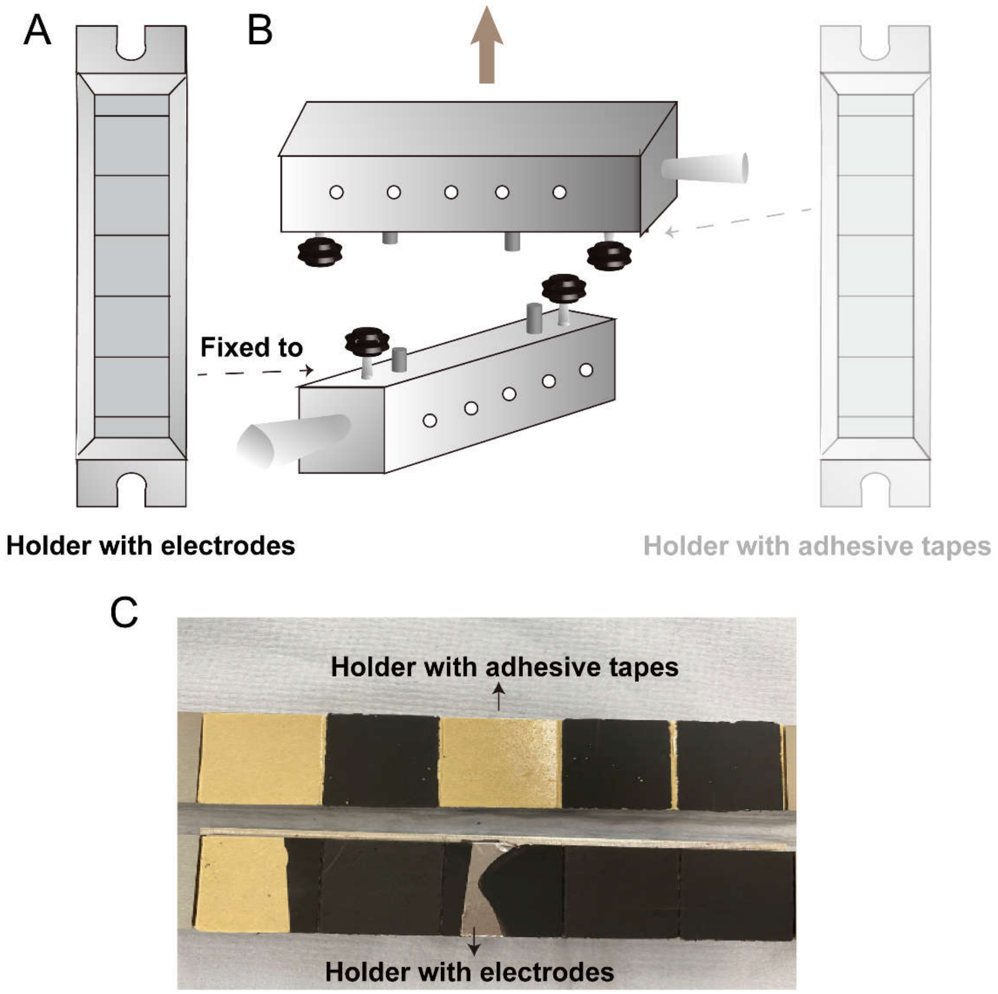

Figure S2. Main parts of the peeling-test device: (A) sample holder for the electrode tapes and (B) two plane parallel plates to fix the holders. The upper plate is movable to peel off the electrode tapes. (C) Photo of the holder with the adhesive tapes (top) and the holder with the electrodes attached to it (bottom) after the peeling test.

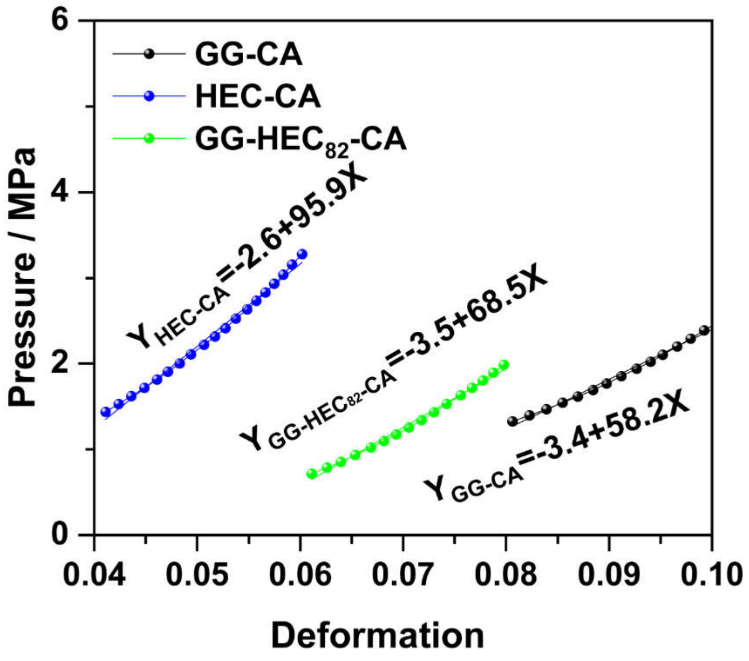

Figure S3. Depiction of the linear fit of the compression test data to determine the modulus.

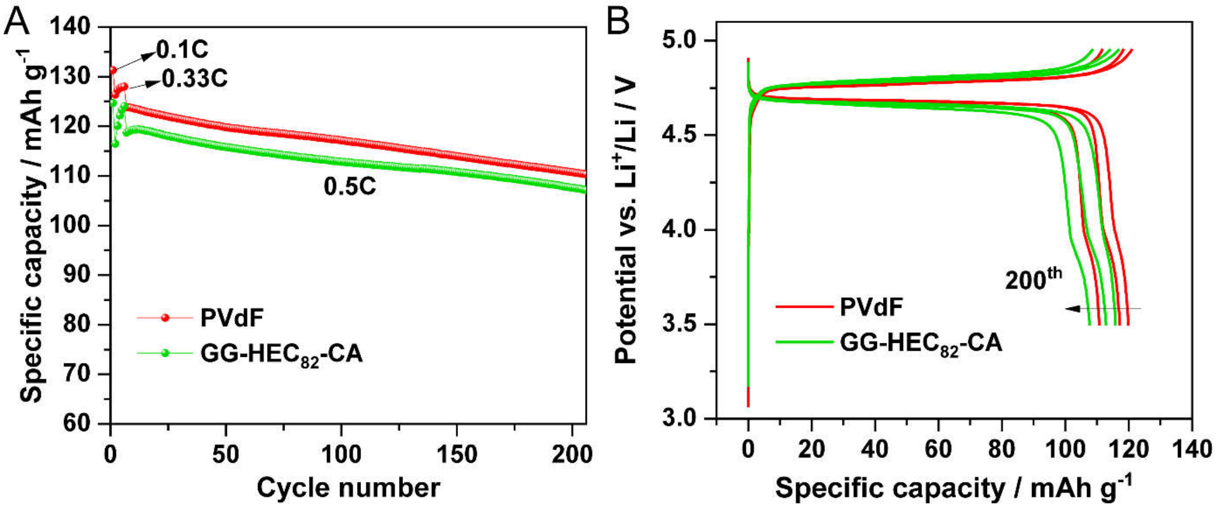

Figure S4. (A) Specific discharge capacity for PVdF and GG-HEC$_{82}$-CA-based LNMO electrodes in Li‖LNMO cells. (B) Selected dis-/charge profiles of the 50th, 100th, and 200th cycle for these cells at a C rate of 0.5C. The cut-off potentials were set to 3.5 and 4.95 V (*vs.* Li^+/Li) and a C rate of 1C corresponds to a specific current of 147 mA g^{-1}.

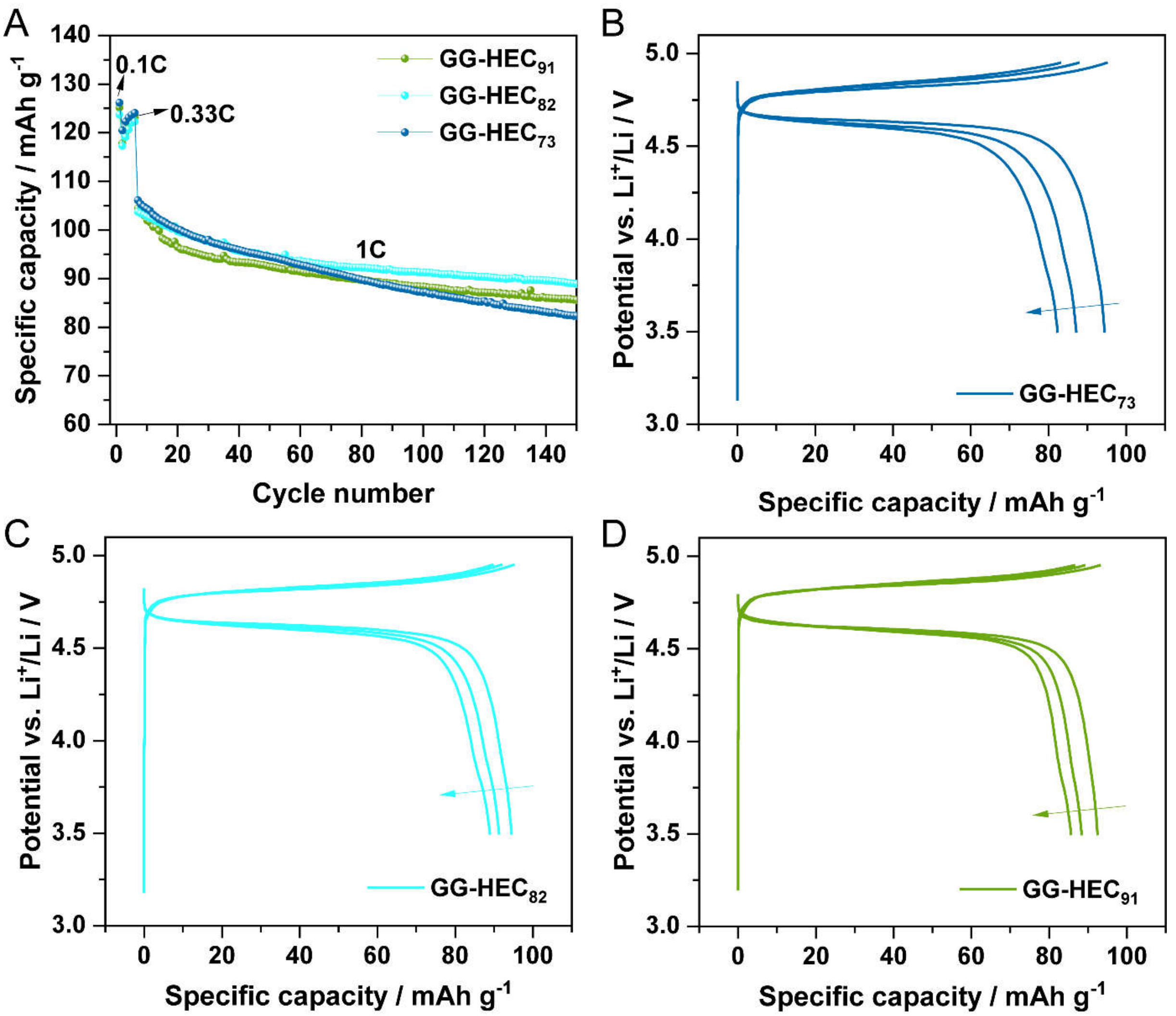

Figure S5. (A) Specific discharge capacity for GG-HEC$_{91}$, GG-HEC$_{82}$, and GG-HEC$_{73}$-based LNMO electrodes upon galvanostatic cycling in Li‖LNMO cells. (B-D) Selected dis-/charge profiles of the 50th, 100th, and 200th cycle for these cells at a C rate of 1C. The cut-off potentials were set to 3.5 and 4.95 V (*vs.* Li^+/Li) and a C rate of 1C corresponds to a specific current of 147 mA g^{-1}.
